# A mild skeletal phenotype with overlapping features of Miller syndrome and functional characterisation of two new variants of human dihydroorotate dehydrogenase

**DOI:** 10.1016/j.heliyon.2024.e38659

**Published:** 2024-09-27

**Authors:** Inger-Lise Mero, Juan Manuel Orozco Rodriguez, Kathrine Bjørgo, Renee Alexandra Hankin, Ewa Krupinska, Mari Ann Kulseth, Marvin Anthony Rossow, Wolfgang Knecht

**Affiliations:** a Department of Medical Genetics, Oslo University Hospital, PB 4956 Nydalen, 0424, Oslo, Norway; b Department of Biology & Lund Protein Production Platform, Lund University, Sölvegatan 35, 22362, Lund, Sweden; c Department of Radiology, Oslo University Hospital, PB 4950 Nydalen, 0424, Oslo, Norway

**Keywords:** Inborn errors of metabolism, Enzymology, Purine and pyrimidine metabolism, Biophysics, Protein production

## Abstract

Dihydroorotate dehydrogenase (DHODH) catalyzes the fourth enzymatic reaction of the pyrimidine biosynthesis pathway. Miller syndrome, also known as postaxial acrofacial dysostosis, is caused by biallelic pathogenic variants in *DHODH*. We present a patient with a relatively mild skeletal phenotype carrying a novel variant of unknown significance in *DHODH*: c.829G > A, p.(D277N), in combination with a known variant, c.403C > T, p.(R135C). We functionally characterized the *DHODH* variant D277N in comparison to a very recently reported, but functionally uncharacterized variant P43L, that was found in a patient with more pronounced Miller syndrome features. Because both cases share the same *DHODH* variant R135C, we aimed to study the effect on enzyme activity of the two variants D277N and P43L to determine pathogenicity and possibly a genotype-phenotype relationship on the R135C background. We found a significant reduction in enzyme activity for both variants. The variant P43L showed a more pronounced loss of function in all assays compatible with other pathogenic variants reported in Miller, whereas the D277N variant showed milder changes that could reflect the mild phenotypic features in our patient. Yet due to a lack of a known threshold of residual enzyme activity to determine pathogenicity, this needs to be confirmed in further studies.

## Highlights

• Identification of a new variant in the *DHODH* gene (c.829G > A, p.(D277N)).
• Description of the relatively mild skeletal phenotype present in the patient.
• Biochemical and biophysical characterization of the new DHODH variant D277N.
• Comparison to a DHODH variant (P43L) from a severe Miller syndrome phenotype.
• Variant P43L fulfilled more criteria for loss of total DHODH activity than D277N.

## 1 Introduction

The *de novo* pyrimidine biosynthesis pathway is nearly universal to all organisms [[1], [2], [3], [4]]. The end-product of this process consisting of six enzymatic steps is uridine monophosphate (UMP). UMP is the starting point for the delivery of deoxynucleoside triphosphate (dNTP) precursors for DNA, nucleoside triphosphate (NTP) precursors for RNA, as well as glycoconjugates and many other metabolically important molecules. The flavoenzyme dihydroorotate dehydrogenase (DHODH) catalyzes the fourth enzymatic reaction of the pyrimidine biosynthesis pathway, the oxidation of dihydroorotate (DHO) to orotate [5,6].

Human DHODH belongs to the Class II of DHODHases, a classification which is based on sequence similarities rather than convergent evolution of different ancestral proteins [7,8]. The enzyme is an integral protein of the inner mitochondrial membrane (IMM) and couples the oxidation of DHO to the reduction of its second substrate, ubiquinone Q_10_, which is found in the center of the IMM [9]. DHODH thereby functionally connects *de novo* pyrimidine biosynthesis to the respiratory chain and their complexes [[10], [11], [12], [13]]. However, the literature suggests that human DHODH is a stand-alone enzyme not associated with any respiratory supercomplexes [14].

Miller syndrome, a skeletal dysplasia also known as postaxial acrofacial dysostosis, is caused by biallelic pathogenic variants in human *DHODH* and became the first Mendelian disorder whose molecular basis was revealed by whole-exome sequencing [15]. The current knowledge about Miller syndrome was recently reviewed by us [16] and the most recent listing of Miller syndrome‐related variants in the *DHODH* gene can be found here [17].

The clinical presentation as originally described by Miller included postaxial limb deficiency, cup-shaped ears, micrognathia and malar hypoplasia in 6/6 patients, whereas additional craniofacial and limb anomalies as well as accessory nipples, were noted in many [18]. The combination of traits in individuals seems to vary considerably, and except for postaxial limb hypo-/aplasia, no single feature has been reported consistently in all patients with a molecularly confirmed diagnosis [15,[19], [20], [21]]. Several of the earlier case studies, including molecularly confirmed and only clinically described patients, mention soft tissue syndactyly between the 1st and 2nd digits and/or hypoplasia of the 1st digit together with underdevelopment of the limbs in general as part of the clinical spectrum [19,21,22].

We present here the description of a patient with relatively mild skeletal phenotype with compound heterozygosity for two variants in the *DHODH* gene. One known pathogenic variant NM_001361.4 c.403C > T, p.(R135C) [15,16,19], and one variant of unknown significance, c.829G > A, p.(D277N).

As the patient phenotype was relatively mild, but still overlapping with features of Miller syndrome, we set out to investigate whether this variant could alter enzyme activity. In our biochemical and biophysical analyses, we also included yet another variant of unknown significance, that is c.128C > T p.(P43L). This variant was recently shown also in combination with R135C in a patient with more pronounced features of Miller syndrome [23], but the variant has not been described functionally. Because we do not know the threshold of residual enzyme activity to determine pathogenicity and both cases share the same *DHODH* variant R135C, we aimed to define the pathogenicity and possibly a genotype-phenotype relationship for those two variants D277N and P43L on the R135C background using an integrated biochemical and biophysical approach in comparison to previous characterisation of wild-type human DHODH performed by us. The effect of the pathogenic *DHODH* variant R135C, which shows very little activity with the natural substrates of DHODH, shared in both cases on a biochemical scale has been characterized by us earlier in detail [16,24].

## 2 Materials and methods

### 2.1 Genetic analyses

Genome sequencing analyses applying a gene panel of 397 genes comprising Genomics England PanelApp “Skeletal dysplasia v 2.9” (https://panelapp.genomicsengland.co.uk), was performed in the proband in a diagnostic setting. We performed targeted Sanger sequencing of the *DHODH* variants in both parents. aCGH 180 K had been performed diagnostically prior to genome sequencing. During the genetic work-up the patient also retrieved a diagnosis of ADHD and autism spectrum disorder (ASD), hence we expanded our analyses to trio genome sequencing applying a panel for developmental disorders and congenital malformation (2522 genes) comprising several panels as described in https://panelapp.genomicsengland.co.uk/ (full panel available on request).

Genome sequencing was performed, applying TruSeq DNA PCR Free preparation followed by NovaSeq 6000 system as delivered by Illumina. Mapping of reads, variant detection and quality control were done by the Illumina DRAGEN™ Bio-IT Platform and in-house scripts. Variants were annotated with Vcfanno [25] and Variant Effect Predictor (VEP) [26]. The variants were classified according to the ACMG criteria [27].

### 2.2 Plasmids

The DHODH D277N and P43L constructs for protein expression in *E. coli* were based on the constructs for wild-type full-length human DHODH described in Ref. [28] and were produced by GenScript (Amsterdam) by site-directed mutagenesis. The plasmids were transformed into chemically competent *E. coli* TUNER(DE3) cells (Novagen).

The DHODH D277N and P43L expression constructs for protein expression in insect cells using the Baculovirus Expression Vector System (BEVS) were based on the constructs for full-length human DHODH as described in Ref. [16] and were also produced by GenScript (Amsterdam) by site-directed mutagenesis.

### 2.3 Protein expression and purification

DHODH D277N and P43L produced in *E. coli* were purified as described in detail in Refs. [16,28] with the following modifications: protein expression was induced by the addition of 10 μM IPTG, and cleavage of the His-tag was performed using TEV protease at a 1:50 TEV:DHODH mass ratio.

### 2.4 Enzyme activity assay

All assays and determinations of kinetic and inhibition constants were done as described in detail in Refs. [16,28]. The concentration of Brequinar was varied in the range from 100 000–0.01 nM and the concentration of Teriflunomide was varied from 500 000–10 nM.

### 2.5 Thermal stability assay by differential scanning fluorimetry (nanoDSF)

Thermal stability assays were performed as described in detail in Ref. [16].

### 2.6 Recombinant expression of DHODHs in insect cells

For production of wildtype DHODH and DHODH D277N or P43L in insect cells, all enzymatic assays and Western Blot analysis with subsequent immunodetection were done with the methods described in detail in Ref. [16]. Comparative expressions of wildtype DHODH, a mock control expressing Green Fluorescent Protein (GFP) and one of the DHODH variants was done each time in parallel.

### 2.7 Statistical analysis

One-way analysis of variance (ANOVA) and *post-hoc* Dunnett's tests were performed with SigmaPlot14 (Systat Software Inc.).

## 3 Results

### 3.1 Clinical patient features

The patient was investigated in the neonatal period due to a cardiac murmur and dysmorphic features including low set ears and bilateral simian creases. Echocardiography was normal except for slight tricuspid insufficiency. Karyotype was normal. At one month of age, he was assessed for eating/swallowing difficulties and episodes of obstructive apnoea during meals and sleep. He had multiple admissions for residual airway infections. pH measurements (24 h) excluded reflux. Nocturnal polysomnography at 7 months showed obstructive sleep apnoea, and he used continuous positive airway pressure (CPAP) during sleep for 9 months. He had language delay, which along with infections and sleep, improved after tonsillectomy at 2 years. At 9 years, he was referred to the clinical genetic department due to a combination of dysmorphic features and attention deficit hyperactivity disorder (ADHD). During the period of genetic work-up he also got a diagnosis of autism spectrum disorder. On inspection, he had low set posteriorly rotated ears with overfolded upper helices and malar flattening. The hands were small with short thumbs, webbed fingers and bilateral simian creases. In the feet there were bilateral sandal gaps, lateral deviation of the second toes, short and broad first toe, and overriding right third/ fourth toe. Height was on the 50th centile**.**

**Fig. 1:**
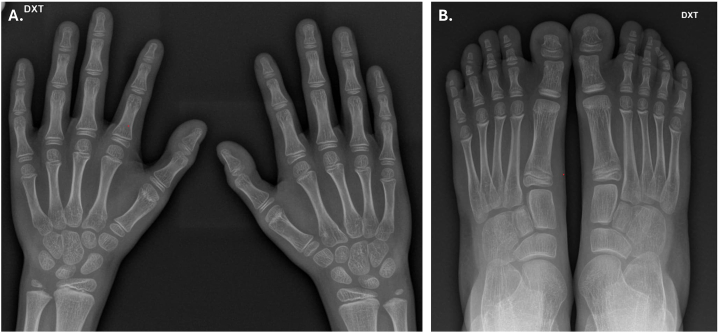
X-ray imaging of the hands (A.) and feet (B.).

X-ray imaging of the hands and feet (Fig. 1) showed subtle, partial soft tissue syndactyly of the 1st and 2nd fingers, and while all phalanges are short compared to age matched normal, the variation is still within normal limits. X-ray imaging of the feet showed partial soft tissue syndactyly of the web between the 1st and 2nd toes. The distal phalanges of the great toe are both short and there are subtly cone-shaped epiphyses of the 2nd – 4th middle phalanges of both feet, these are also on the short side compared to age matched normal, however cone-shaped epiphyses can be a normal variant. The distal phalanges of the 2nd and 5th fingers gave an impression of a slightly triangular shape.

### 3.2 Genetic analyses

Genome sequencing revealed heterozygosity for two variants in the *DHODH* gene:

(NM_001361.4); c.403C > T, p.(R135C) and c.829G > A, p.(D277N). Parental analyses confirmed compound heterozygosity of the variants. The variant c.403C > T has previously been shown in multiple patients with Miller syndrome and functional analyses have confirmed its severely reduced function [16,24]. This variant has a population carrier frequency (heterozygous only) of 0,06 % according to gnomAd v4 (https://gnomad.broadinstitute.org/). The Revel score was 0,901 fulfilling moderate evidence for pathogenicity [29].

The c.829G > A, p.(D277N), has a frequency of 0.002 % according to gnomAd v4 (heterozygotes only). The revel score was 0.723 fulfilling supporting evidence for pathogenicity [29]. Both amino acids R135 and D277 are conserved in all sequenced species, from primate to fish using the Vertebrate multiz alignments in the UCSC browser (https://genome.ucsc.edu/) comparing 100 species. However, when broadening the evolutionary range of Class II DHODHases as done in a recent comprehensive bioinformatics study by Sousa et al. [30] using 1062 Class II DHODH sequences, D277 is not among the amino acid residues present in at least 80 % of those sequences in contrast to R135 which is highly conserved.

### 3.3 Location of the mutations

**Fig. 2:**
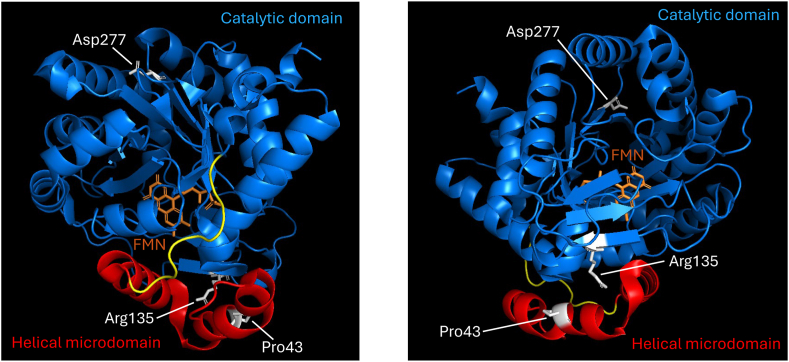
Location of the point mutations D277N andP43L, as well as R135C in the human DHODH variants. The figure shows the location of the mutations in a molecular model of human DHODH based on the crystal structure of the soluble domains of DHODH (PDB entry 1d3h). For better visibility of the residues, the structure is shown in 2 orientations. The catalytic domain is indicated in blue. The α1-α2 microdomain is shown in red. The FMN cofactor is displayed in orange. The locations of the residues D277, P43 and R135 are indicated in grey. Please note that this structure does not contain the mitochondrial signal sequence, nor the transmembrane domain that anchors and orients DHODH in the IMM.

The mutations P43L or D277N in the human DHODH variants are in the membrane-interacting α1-α2 microdomain and the catalytic domain, respectively, as shown in Fig. 2. An obvious functional relevance could not be assigned to them on the basis of the bioinformatic analysis of Class II DHODHases in Ref. [30] and its underlying structural information.

### 3.4 Protein expression and purification

DHODH D277N and P43L were produced as full-length enzymes as described for wild-type full-length human DHODH [16,28]. DHODH D277N expressed well in *E. coli* cells. There were no signs of aggregation or precipitation during the purification procedure. The yield of pure enzyme per liter of bacterial culture amounted to 10.8 mg/L. DHODH P43L showed much less total activity and yielded only about 0.5 mg per L cell culture volume. The respective purification table and corresponding SDS-PAGE analysis can be found in the supplementary material as Table S1 and Fig. S1.

### 3.5 Kinetic characterisation of the purified DHODH mutants D277N and P43L

We first determined the kinetic constants with the standard activity assay for DHO and the ubiquinone analogue decylubiquinone (Q_D_) which is mostly used throughout the literature [10,28,31] due to its solubility in micelles as well as in the solution phase. We also used the natural substrate of DHODH's ubiquinone Q_10_ and the only water-soluble ubiquinone, benzoquinone (Bz), an analogue which lacks the side chain, [16,24]. The results are presented in Table 1 and compared to wild-type human DHODH previously characterized by us with the same methods [16].

**Table 1:** Kinetic parameters of wild-type DHODH and Miller syndrome variants. The maximum rate (V_max_) and the Michaelis-Menten constant (K_m_) of these enzymes with respect to the substrates (DHO and ubiquinone) are presented. The values represent the average ± SD calculated from 3 to 5 independent measurements. For determination of the kinetic constants, DHO was varied from 0 to 1 mM while Q_D_ was kept constant at 0.1 mM. Alternatively, Q_D_ or Q_10_ was varied from 0 to 0.1 mM while DHO was held constant at 1 mM. On the other hand, Benzoquinone (Bz) was varied from 0 to 1 mM while DHO was held constant at 1 mM. Values that are significantly different (P < 0.05) with respect to the wild type DHODH according to one-way analysis of variance (ANOVA) and post-hoc Dunnett's test are indicated by an asterisk (∗).

Enzyme	DHO^a^			Q_D_^a^			Q_10_^a^			Bz		
	K_m_ (μM)	V_max_ (U/mg)	k_cat_/K_m_ ( × 10^6^ s^−1^ M^−1^)	K_m_ (μM)	V_max_ (U/mg)	k_cat_/K_m_ ( × 10^6^ s^−1^ M^−1^)	K_m_ (μM)	V_max_ (U/mg)	k_cat_/K_m_ ( × 10^6^ s^−1^ M^−1^)	K_m_ (μM)	V_max_ (U/mg)	k_cat_/K_m_ ( × 10^6^ s^−1^ M^−1^)
DHODH^a^	16.0 ± 4.9	122.8 ± 13.6	5.9 ± 2.0	20.3 ± 2.7	129.9 ± 3.2	4.6 ± 0.6	7.5 ± 1.3	9.5 ± 1.2	0.9 ± 0.3	117.5 ± 24	21.1 ± 3.8	0.13 ± 0.006
DHODH D277N	16.5 ± 2.2	74.9 ± 2.9 ∗	3.4 ± 0.4	4.6 ± 1.1 ∗	70.3 ± 1.5 ∗	11.7 ± 2.6 ∗	16.7 ± 8.5	3.6 ± 0.6 ∗	0.18 ± 0.077 ∗	58.5 ± 15 ∗	8.4 ± 0.6 ∗	0.11 ± 0.02
DHODH P43L	13.9 ± 2.1	32.0 ± 2.1 ∗	1.7 ± 0.3 ∗	2.4 ± 0.4 ∗	35.2 ± 12.8 ∗	10.2 ± 2.9 ∗	10 ± 7.4	1.7 ± 1.2 ∗	0.15 ± 0.074 ∗	375.6 ± 33 ∗	23.8 ± 1.8	0.047 ± 0.005 ∗

a Results from [16].

With respect to catalytic efficiency for the two natural substrates, DHO and Q_10_, DHODH P43L showed a significant decrease in catalytic efficiency towards both substrates whereas in the case of DHODH D277N the decrease was only observed for Q_10_. This change in catalytic efficiency was in both variants caused by a reduction in V_max_ with DHODH P43L showing a more pronounced decrease in V_max_ than DHODH D277N. Interestingly, the catalytic efficiency with the substrate Q_D_ increased significantly for both variants, caused by a significant improvement of K_m_ for this substrate even on the background of decreased V_max_ values. When testing the only water-soluble ubiquinone analog Bz, only DHODH P43L showed a significant decrease in catalytic efficiency.

### 3.6 Inhibition of the purified DHODH mutants D277N and P43L by Brequinar and Teriflunomide

Brequinar and Teriflunomide are known to bind in human DHODH in a tunnel like location through which it is assumed that ubiquinone approaches the FMNH_2_ cofactor [[16], [28]]. Therefore, Brequinar and Teriflunomide can be used as probes to detect possible perturbations in this tunnel caused by mutations in DHODH [16]. The results are presented in Table 2 and compared to wild-type human DHODH previously characterized by us with the same methods [16].

**Table 2:** Half maximal inhibitory concentration (IC_50_) for Brequinar and Teriflunomide. The IC_50_ reported are the average of 3–4 independent measurements ± SD. The average of the fitted slopes ± SD are given in brackets. IC_50_ values that are significantly different from the IC_50_ value with DHODH (*P* < 0.05) as determined by one-way analysis of variance (ANOVA) and post-hoc Dunnett's test are indicated by an asterisk (∗).

Enzyme	IC_50_ (nM)	
	Brequinar	Teriflunomide
DHODH^a^	4.3 ± 0.9 (1.16 ± 0.17)	1094 ± 347 (0.88 ± 0.04)
DHODH D277N	7.1 ± 1.4 (0.89 ± 0.20)	741 ± 89 (0.76 ± 0.06)
DHODH P43L	134.9 ± 36.9 ∗ (0.85 ± 0.25)	2954 ± 107 ∗ (0.95 ± 0.11)

a Results from [16].

The IC_50_ values of Brequinar and Teriflunomide with respect to the DHODH D277N variant were not significantly different from those determined for the wild-type enzyme. The IC_50_ values for the DHODH P43L were significantly higher than for the wild-type protein showing less affinity of the compounds for this variant.

### 3.7 Thermal stability of the purified DHODH mutants D277N and P43L

Besides changes in the catalytic efficiency of the mutants, changes in protein stability have been shown for mutants associated with Miller syndrome [16]. We therefore tested the thermal stability the two mutant enzymes and whether they could be stabilized by binding to Brequinar or Teriflunomide (Table 3). The results were again compared to wild-type human DHODH previously characterized by us in the same way [16].

**Table 3:** Thermal stability data determined by differential scanning fluorimetry.

Enzyme	T_m_ (°C)		
	Buffer + DDM	Buffer + DDM + Brequinar	Buffer + DDM + Teriflunomide
DHODH^a^	57.87 ± 1.69	75.77 ± 1.70	68.00 ± 0.40
DHODH D277N	59.3 ± 0.08	79.2 ± 0.10 ∗	74.1 ± 0.08 ∗
DHODH P43L	51.4 ± 0.07 ∗	71.3 ± 0.20 ∗	67.4 ± 0.07 ∗

The melting temperature (average ± SD) was calculated from 3 independent measurements. The buffer used was 10 mM Tris-HCl, 100 mM NaCl, pH 7.4. DDM was used at 0.6 mM (5 times the critical micellar concentration). Brequinar was used at 100 μM and Teriflunomide was used at 500 μM. T_m_ values of the mutants that are significantly different (P < 0.05) with respect to the wild type DHODH according to one-way analysis of variance (ANOVA) and post-hoc Dunnett's test are indicated by an asterisk (∗).

a Results from [16].

The T_m_ values of the DHODH D277N mutant were comparable to those of the wild-type enzyme. However, in the presence of both Brequinar and Teriflunomide they were significantly higher than those of the wild-type protein (ΔT_m_ = + 3.4 °C for Brequinar and ΔT_m_ = + 6.8 °C with Teriflunomide.). On the other hand, DHODH P43L was less stable than the wild-type enzyme and it was not stabilized to the same level as the wild-type enzyme in the presence of high concentrations of Brequinar or Teriflunomide.

### 3.8 Expression of DHODH mutants D277N and P43L in insect cells

We then used the same approach outlined in Ref. [16] to characterize the mutants in a eukaryotic cell environment. While the most efficient source for high yields of purified recombinant protein needed for kinetic and, in particular, biophysical characterization, is *E. coli*, we also expressed all mutants in insect cells using the BEVS. This is based on previous work showing that DHODH is found in mitochondria and characterizations can be done taking advantage of its native localization [10,16].

**Fig. 3:**
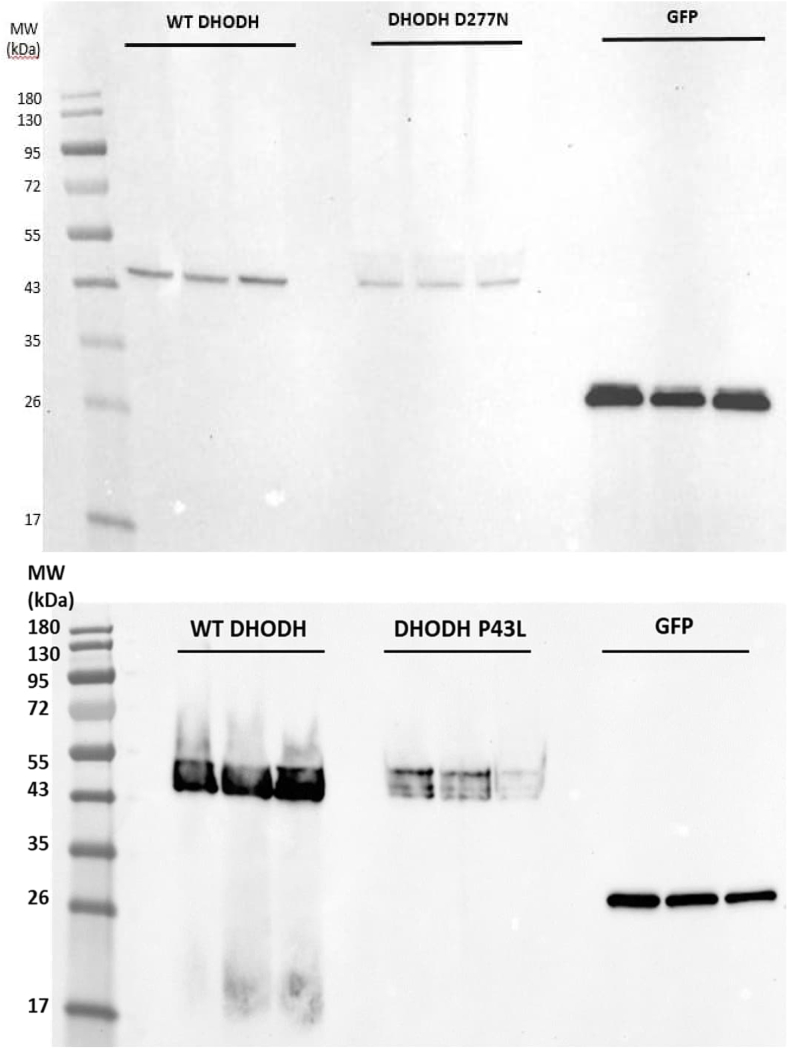
Western blotting and immunodetection of the expression of wild-type DHODH, DHODH D277N and P43L in Sf9 insect cells at 72 h post infection. One of three to four Western blots is displayed, showing three biological replicates for each infection. Wild-type and mutant DHODH were detected with an anti-His antibody. Mock infection was done with a virus expressing GFP.

We infected Sf9 insect cells with recombinant baculovirus expressing either wild-type DHODH, the two DHODH variants or GFP as background control and analysed the whole cell extracts 72 h after infection for DHODH enzyme activity with 1 mM DHO and 0.1 mM Q_D_ (Table 4) as well as for DHODHase expression levels by Western blotting and immunodetection (Fig. 3). These experiments were done separately for DHODH D277N and P43L at different times in the project, but each time in parallel to expression of wild-type human DHODH and GFP. They are therefore separately presented in Table 4.

**Table 4:** Specific activity of DHODH in whole cell extracts of insect cells expressing DHODH and Miller syndrome mutants measured 72 h after infection.

Enzyme	Specific activity (%)	Signal strength on blot (%)	Specific activity (%)	Signal strength on blot (%)
DHODH	100	100	100	100
DHODH D277N	90.1 ± 41.2	85.1 ± 57.3		
DHODH P43L			10.4 ± 3.5	34 ± 19
Sf9 cells (GFP expression)	16.0 ± 1.7	NA	0.13 ± 0.12	NA

In each measurement the specific activity of DHODH was set to 100 %. DHO was kept at 1 mM and Q_D_ at 0.1 mM. The average activities ± SD of 3 measurements from 3 separate cultures are reported. Additionally, we show the relative signal strength on Western blots. The average signal strength measured on three blots of the three expression cultures ± SD is shown. The signals represent the intensity relative to that of wild-type DHODH, which was set at 100 % for each blot. NA – not applicable.

For DHODH D277N expression in the insect cells, the specific activity measured for DHO in combination with the ubiquinone analogue Q_D_ did not differ from that of wild-type enzyme, neither did we detect less expression levels than for the wild-type. On the contrary, cell extracts from the DHODH P43L expression showed lower levels of specific activity and less DHODH expression.

To test the activity of the mutant enzymes in their natural environment without any addition of detergents or quinones, we used the assays originally described by Fang et al. [32] for DHODH in whole cell extracts with further modifications described by us [16]. We also measured another mitochondrial enzyme, succinate dehydrogenase (SDH) as baseline and to demonstrate that the cell extracts are of comparable quality. For the same reasons as explained above, they are again separately presented in Table 5.

**Table 5:** Specific DHODH and succinate dehydrogenase (SDH) activities in Sf9 insect cell extracts expressing DHODH and Miller syndrome mutants in the absence of detergents or additional electron acceptors.

Enzyme	DHODH (%)	SDH (%)	DHODH (%)	SDH (%)
DHODH	100 ± 33.3	100 ± 11.4	100 ± 32.4	100 ± 7.9
DHODH D277N	33.3 ± 13.3 ∗	113.9 ± 20.3		
DHODH P43L			15.2 ± 5.2 ∗	110.2 ± 17.3
Sf9 cells (mock infection)	4 ± 1.3 ∗	88.6 ± 11.4	1 ± 1 ∗	92.2 ± 3.6

Either 20 mM DHO or 20 mM succinate was used as the substrate, respectively. Activities were calculated in nmol of substrate converted per minute per mg of total protein (u/mg) at 37 °C. Average ± SD was calculated from 3 independent measurements. For easier comparison, the average specific activities in wildtype DHODH expressions were set to 100 % in this table. Specific activities that are significantly different (P < 0.05) with respect to the activities in extracts from wildtype DHODH expression according to one-way analysis of variance (ANOVA) and post-hoc Dunnett's test are indicated by an asterisk (∗).

No significant differences in SDH activity between the different cell extracts could be detected suggesting that their preparation delivered samples of comparable quality for mitochondrial content based on activity of the endogenous mitochondrial enzyme SDH. However, for both variants a significant drop in DHODHase activity could be measured, with a stronger decline in activity of the DHODH P43L variant than for the D277N variant.

## 4 Discussion

We have identified a novel *DHODH* variant, D277N, and recently, another Miller syndrome patient with a P43L variant and pronounced Miller syndrome phenotype was reported [23]. Both patients share the same second *DHODH* variant R135C, but they show very different phenotypes. The patient phenotype presented in our study now was relatively mild, with some overlapping skeletal features of Miller syndrome; in particular the malar flattening and webbing between the 1st and 2nd digits, the shortened 1st digits of the feet and the subtle skeletal developmental delay, especially of the hands. While these features are consistent with the known phenotypic skeletal manifestations displayed by patients with genetically confirmed Miller's syndrome, they are not exclusive to this diagnosis radiologically. Furthermore, the patient had neurobehavioral diagnoses, which are discordant with the phenotypic description of Miller syndrome. To the best of our knowledge, no other patient has been reported before with two *DHODH* variants and a mild skeletal phenotype like our patient.

We have therefore characterized and compared the two *DHODH* variants using biochemical and biophysical methods to determine whether they are likely pathogenic and whether relative loss of enzymatic activity could correlate to the severity of the phenotype. The potential decrease of total enzymatic activity for human DHODH can be caused by a) a decrease in the efficiency of use of its substrates b) changed interaction with the lipids of the IMM impairing catalytic efficiency c) an impaired mitochondrial import d) a decreased stability [16,24]. Points c and d would result in a decrease of the total number of active enzymes, even if a) and b) would not be affected. Our key findings with respect to usage of the natural substrates of DHODH and thermal stability are summarized in Table 6. We also include in this table the previously published data [16] on the shared second *DHODH* variant R135C of both cases.

**Table 6:** Summary of the key differences found in our study.

	DHODH D277N	DHODH P43L	DHODH R135C^a^
DHO V_max_	61 ∗	27 ∗	2 ∗^a^
DHO k_cat_/K_m_	58	29 ∗	5 ∗^a^
Q_10_ V_max_	38 ∗	18 ∗	7 ∗^a^
Q_10_ k_cat_/K_m_	20 ∗	16 ∗	2 ∗^a^
Thermal stability (T_m_ value)	102	89 ∗	103^a^
Total DHODH activity in whole cell extract with endogenous electron acceptor	33 ∗	15 ∗	15 ∗^a^

Combination of DHODH variants	D277N/R135C	P43L/R135C	R135C/R135C
Miller Syndrome phenotype	mild (this study)	typical [23]	no cases identified

Values are given in % of wild-type DHODH. Statistically significant differences are indicated by an asterisk (∗). Data are taken from Table 1, Table 3, Table 5 as well as ^a^ results from Ref. [16]. The lower part of the table specifies the phenotype of the possible combinations of the three variants.

Table 6 clearly shows that both isolated enzyme variants have a statistically significant reduced V_max_ for both DHO and Q_10_ as well as for the catalytic efficiency with which Q_10_ is utilized. We can therefore conclude that both variants show a significantly decreased catalytic efficiency in the usage of one or both natural substrates. In comparison, the previously characterized variant R135C shows very little activity with the natural substrates of DHODH.

P43L is in the α1-α2 N-terminal microdomain of DHODH and causes a thermal destabilisation of the enzyme, while D277N is as stable as the wild-type enzyme. In this respect, P43L is comparable to another variant related to Miller syndrome patients, c.155A > G, p.(E52G), which is also located in the α1-α2 N-terminal microdomain of DHODH, and which we recently found to destabilize human DHODH [16]. E52 is an amino acid highly conserved in all Class II DHODHases [30]. While DHODH P43L shows 11 % decrease in T_m_, E52G shows a decrease of 30 % [16]. The decreased thermo-stability is consistent with, and probably also the explanation for the lower expression level of DHODH P43L (Table 4 & Fig. 3) we observed when expressing it in insect cells, as well as the low yields for the purified recombinant enzyme (Table S1). The same pattern was previously observed for DHODH E52G [16]. DHODH D277N on the other hand behaved like wild-type DHODH in these experiments.

In whole cell extracts without addition of exogenous ubiquinone electron acceptor or detergents, the total enzyme activity of DHODH D277N drops to 33 % compared to wild-type DHODH and the activity of DHODH P43L decreases to 15 %. We previously reported total activity drops for the Miller Syndrome related DHODH variants c.56G > A, p.(G19E), c.155A > G, p.(E52G) and c.403C > T, p.(R135C) to activity levels corresponding to 24 %, 20 % and 15 % of the wild-type DHODH activity, respectively [16]. In this context, the DHODH variant D277N shows the highest residual activity level. In summary, DHODH P43L fulfils more criteria for potential loss of total enzyme activity (Table 6) than DHODH D277N and in all experiments shows a more pronounced loss of function. Altogether, the above indicates, that the Miller phenotype reported by Ref. [23] reflects the activity of the *DHODH* variant P43L which is comparable to other pathogenic variants reported in Miller syndrome. The mild phenotype observed in our case could possibly reflect the decreased activity of the *DHODH* variant D277N, but this genotype-phenotype relationship needs confirmation of further cases with mild phenotypes.

Regarding what can be said about the structural changes on the atomic scale, we can assume that some structural changes do take place, and pinpoint one location based on our biochemical characterisation, the hydrophobic tunnel through which ubiquinone should access the FMN co-factor. For DHODH P43L the binding of both Brequinar and Teriflunomide in this location is impaired even if the mutation is not near their binding site. Another Miller syndrome variant has been reported with greatly increased IC_50_ values for Brequinar and Teriflunomide: DHODH R135C [16]. R135 (Fig. 2) is known to make key binding contacts with both inhibitors and therefore this is not surprising. The hypothesis of structural changes in the access tunnel for Q_10_ towards FMN is supported by the changes in kinetic parameters for both Q_10_ and Q_d_ that can access DHODH from the micelles in which purified full-length DHODH is found [24]. Further changes in the proximity between purified DHODH and the micelles is implied by the changes in kinetic parameters of Bz, a ubiquinone analogue that can access DHODH only from the aqueous phase [24]. In summary, this indicates structural changes in the variants and changed interactions with hydrophobic micelles.

A question raised for Miller syndrome is if and what connections we can make between remaining residual enzymatic activity of DHODH and the severity of the phenotype of a patient [19]. This is a common problem with many autosomal recessive disorders: the threshold of residual enzyme activity to determine whether there is pathogenicity or not, is not known. Mice homozygous for a knock-out allele exhibit abnormalities in trophoblast layer formation and complete embryonic lethality during organogenesis (https://www.informatics.jax.org/marker/MGI:1928378, accessed 2024-01-17). It is therefore reasonable to assume that the threshold for a recognisable Miller syndrome phenotype should be somewhere between 50 % and 0 % total enzymatic activity compared to an individual homozygous for wildtype *DHODH*. The variant R135C, the common background in both cases discussed here, retains little activity *in vitro*, but to our knowledge no patient has been described with this variant in homozygous form, but always in compound heterozygosity with another DHODH variant and never in a mild phenotype [15,19,23]. This would be consistent with the very low activity of the variant R135C and making cases of homozygous occurrence not compatible with life. However, an ultimate assignment of the relative contribution of each variant in compound heterozygotes to the phenotype has still to be regarded as a hypothesis until more DHODH variants in further cases and controls can be studied.

This uncertainty also remains, because studies on Miller syndrome variants, including ours, suffer from the lack of DHODH activity measurements in patient, parental and control samples. Most characterisations of DHODH variants have been done using biochemical and biophysical methods as we do here [19,32]. These studies have sometimes been combined with efforts to find an easy to determine biomarker for Miller syndrome. However, to our knowledge no biomarker has been found so far consistent in patients hitherto investigated, that could strengthen the diagnosis of Miller syndrome, as exemplified by DHO levels [19,33]. We had unfortunately no access to methods for the determination of DHO or orotate levels. We therefore argue based on the parameters available to us, since our patient has a mild skeletal phenotype, and even discordant features of the diagnosis, that this hampers the certainty of a clinical diagnosis of Miller syndrome. We also note that micrognathia, a common feature for patients with a molecularly confirmed diagnosis involving combined heterozygosity for R135C [23] was not seen in our patient.

We do, however, see the combination of craniofacial and skeletal anomalies in the hands, which are hallmarks of Miller syndrome.

## 5 Conclusions

We provide functional evidence for a more pronounced enzymatic loss of function of the *DHODH* P43L variant earlier reported in a patient with Miller syndrome phenotype [23] compared to the D277N variant seen in our patient with a mild phenotype, both in combination with the R135C variant. We argue that the P43L is a likely pathogenic variant with respect to Miller syndrome. Even if the milder change observed in the case of the D277N variant could reflect a milder phenotype, we believe that DHODH variants in further cases and controls must be studied to define an enzymatic threshold to cause a skeletal phenotype.

## Ethical approval

The study was approved by the Norwegian Ethical Committee (REK 2010_1152 with date of 2010-11-19 and update 2019-03-26) and the parents have given their written informed consent to the publishing of findings. We thereby followed local laws on the age and circumstances under which minors may consent for themselves.

## Data availability statement

Data will be made available on request, however access to the full patient journal cannot be given.

## Funding

We thank Lund University, the Royal Physiographic Society of Lund, the Erik Philip-Sörensen Foundation, and the Jörgen Lindström Foundation for financial support.

## CRediT authorship contribution statement

**Inger-Lise Mero:** Writing – review & editing, Writing – original draft, Project administration, Investigation, Formal analysis, Data curation, Conceptualization. **Juan Manuel Orozco Rodriguez:** Writing – review & editing, Writing – original draft, Visualization, Validation, Investigation, Formal analysis, Data curation. **Kathrine Bjørgo:** Writing – review & editing, Investigation. **Renee Alexandra Hankin:** Writing – review & editing, Investigation. **Ewa Krupinska:** Writing – review & editing, Investigation. **Mari Ann Kulseth:** Writing – review & editing, Investigation. **Marvin Anthony Rossow:** Writing – review & editing, Validation, Investigation, Data curation. **Wolfgang Knecht:** Writing – review & editing, Writing – original draft, Visualization, Validation, Supervision, Resources, Methodology, Funding acquisition, Formal analysis, Data curation, Conceptualization.

## Declaration of competing interest

The authors declare that they have no known competing financial interests or personal relationships that could have appeared to influence the work reported in this paper.
