# Supplementary material for: A mild skeletal phenotype with overlapping features of Miller syndrome and functional characterisation of two new variants of human dihydroorotate dehydrogenase
Source: Heliyon. 2024 Sep 27;10(19):e38659. doi: 10.1016/j.heliyon.2024.e38659 (PMC11489341; doi:10.1016/j.heliyon.2024.e38659)
Supplement: Multimedia component 1 [file mmc1.pdf]

Supplementary material

A mild skeletal phenotype with overlapping features of Miller syndrome and functional characterisation of two new variants of human dihydroorotate dehydrogenase

Inger-Lise Mero[1#*], Juan Manuel Orozco Rodriguez[2#], Kathrine Bjørgo[1], Renee Alexandra Hankin[3], Ewa Krupinska[2], Mari Ann Kulseth[1], Marvin Anthony Rossow[2], Wolfgang Knecht[2*]

[1] Department of Medical Genetics, Oslo University Hospital, PB 4956 Nydalen, 0424 Oslo, Norway (I.M., uxwmin@ous-hf.no; K.B., uxatjr@ous-hf.no; M.A.K., makulset@ous-hf.no)

[2] Department of Biology & Lund Protein Production Platform, Lund University, Sölvegatan 35, 22362 Lund, Sweden (J.M.O.R., manuel.orozco@biol.lu.se; E.K., ewa.krupinska@biol.lu.se; M.A.R., rossow-m@web.de; W.K., Wolfgang.knecht@biol.lu.se)

[3] Department of Radiology, Oslo University Hospital, PB 4950 Nydalen, 0424 Oslo, Norway (R.A.H., b32826@ous-hf.no)

[#] These authors contributed equally.

Correspondence: Wolfgang Knecht, Department of Biology & Lund Protein Production Platform, Lund University, Sölvegatan 35, 22362 Lund, Sweden (wolfgang.knecht@biol.lu.se, +46 462227785); Inger-Lise Mero, Department of Medical Genetics, Oslo University Hospital, PB 4956 Nydalen, 0424 Oslo, Norway (uxwmin@ous-hf.no, +47 23075716).

Table S1. Representative purification table for human DHODH D277N purified from 8 L of bacterial culture and human DHODH P43L purified from 6 L bacterial culture.

Purification step	Total activity (U)	Total protein (mg)	Specific activity (U/mg)	Purification factor	Activity yield (%)
**DHODH D277N**					
Cell lysis	42156	9555	4.4	1.0	100
Clarification of lysate	37524	7900	4.8	1.1	89
First IMAC	7101	305	23.3	5.3	17
His-tag removal and second IMAC	7699	136	56.5	12.8	18
Size exclusion chromatography	6068	86	70.6	16.0	14
**DHODH P43L**					
Cell lysis	1168	6400	0.18	1	100
Clarification of lysate	1157	4600	0.25	1.4	99
First IMAC	373	50	7.5	40.9	32
His-tag removal and second IMAC	290	17	17.1	93.5	25
Size exclusion chromatography	68	3.1	22.1	121.2	5.8

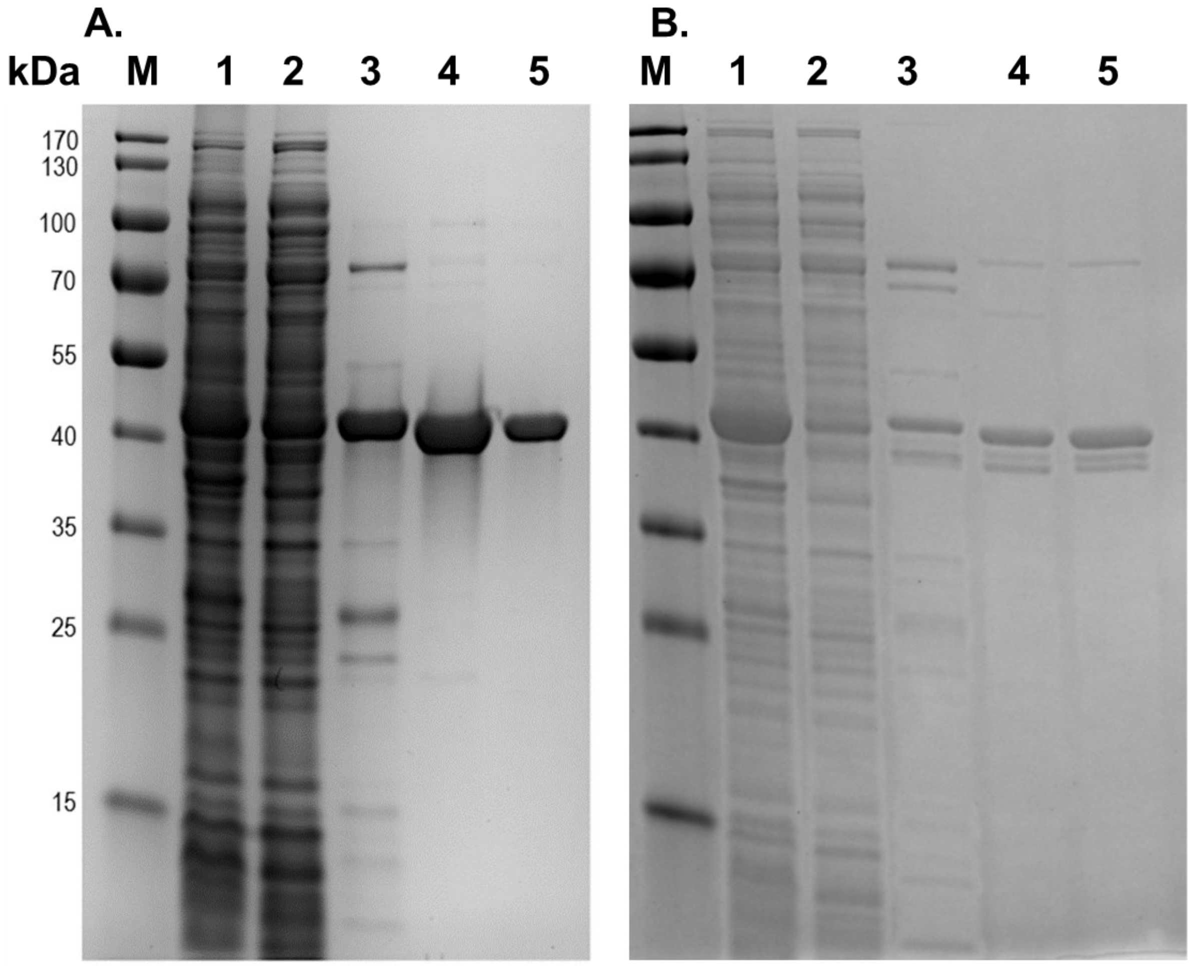

Figure S1. SDS-PAGE analysis of the purification of human A. DHODH D277N and B. DHODH P43L. The proteins in the gels were stained with Coomassie brilliant blue. Lane M, molecular mass marker. Lane 1, cell lysate. Lane 2, soluble fraction. Lane 3, eluate from first IMAC. Lane 4, protein after His-tag removal and second IMAC. Lane 5, protein after size exclusion chromatography (2 µg per lane).
